# A case of allergic bronchopulmonary aspergillosis successfully treated with mepolizumab

**DOI:** 10.1186/s12890-018-0617-5

**Published:** 2018-03-27

**Authors:** Takeshi Terashima, Taro Shinozaki, Eri Iwami, Takahiro Nakajima, Tatsu Matsuzaki

**Affiliations:** Department of Respiratory Medicine, Tokyo Dental College Ichikawa General Hospital, 5-11-13, Sugano, Ichikawa, Chiba 272-0824 Japan

**Keywords:** Mepolizumab, Allergic bronchopulmonary aspergillosis, Bronchial asthma, Eosinophilia

## Abstract

**Background:**

Allergic bronchopulmonary aspergillosis (ABPA) is an allergic pulmonary disease comprising a complex hypersensitivity reaction to *Aspergillus fumigatus*. Clinical features of ABPA are wheezing, mucoid impaction, and pulmonary infiltrates. Oral corticosteroids and anti-fungal agents are standard therapy for ABPA, but long-term use of systemic corticosteroids often causes serious side effects.

**Case presentation:**

A 64-year-old woman was diagnosed with ABPA based on a history of bronchial asthma (from 40 years of age), elevated total IgE, the presence of serum precipitating antibodies and elevated specific IgE antibody to *A. fumigatus*, and pulmonary infiltration. Bronchoscopy showed eosinophilic mucoid impaction. Systemic corticosteroid therapy was initiated, and her symptoms disappeared. Peripheral eosinophilia and pulmonary infiltration recurred five months after cessation of corticosteroid treatment. Systemic corticosteroids were re-initiated and itraconazole was added as an anti-fungal agent. The patient was free of corticosteroids, aside from treatment with a short course of systemic corticosteroids for asthma exacerbation, and clinically stable with itraconazole and asthma treatments for 3 years. In 2017, she experienced significant deterioration. Laboratory examination revealed marked eosinophilia (3017/μL) and a chest computed tomography (CT) scan demonstrated pulmonary infiltration in the left upper lobe and mucoid impaction in both lower lobes. The patient was treated with high-dose inhaled corticosteroid/long-acting beta-agonist, a long-acting muscarinic antagonist, a leukotriene receptor antagonist, and theophylline; spirometry revealed a forced expiratory volume in 1 s (FEV_1_) of 1.01 L. An uncontrolled asthma state was indicated by an Asthma Control Test (ACT) score of 18. Mepolizumab, 100 mg every 4 weeks, was initiated for the treatment of severe bronchial asthma with ABPA exacerbation. Bronchial asthma symptoms dramatically improved, and ACT score increased to 24, by 4 weeks after mepolizumab treatment. Peripheral eosinophil count decreased to 174/μL. Spirometry revealed improvement of lung function (FEV_1_: 1.28 L). A chest CT scan demonstrated the disappearance of pulmonary infiltration and mucoid impaction.

**Conclusions:**

To our knowledge, this is the first case of ABPA to be treated with mepolizumab. Dramatic improvements were observed in symptoms, lung function, peripheral eosinophil counts, and chest images. Mepolizumab could serve as an alternative treatment with the potential to provide a systemic corticosteroid-sparing effect.

## Background

Allergic bronchopulmonary aspergillosis (ABPA) is an allergic pulmonary disease with a complex hypersensitivity reaction to *Aspergillus fumigatus*. Clinical characteristics of ABPA include recurrent asthma exacerbations; chest images of affected patients reveal mucoid impaction, pulmonary eosinophilic infiltrates, and bronchiectasis. Allergic immune reactions can be observed, such as peripheral eosinophilia and elevated total IgE, as well as the presence of serum precipitating antibodies and elevated specific IgE antibody to *A. fumigatus* [1, 2]. Because ABPA is caused by a hypersensitivity reaction to bronchial colonization by *A. fumigatus*, standard therapy comprises a combination of systemic corticosteroids (to attenuate allergic inflammation) and anti-fungal agents (to reduce the fungal load). Serious side effects of corticosteroids are a notable risk in patients undergoing long-term treatment. In this report, we describe a case of severe bronchial asthma with ABPA that was successfully treated with mepolizumab, a recombinant anti-IL-5 antibody.

## Case presentation

**Fig. 1:**
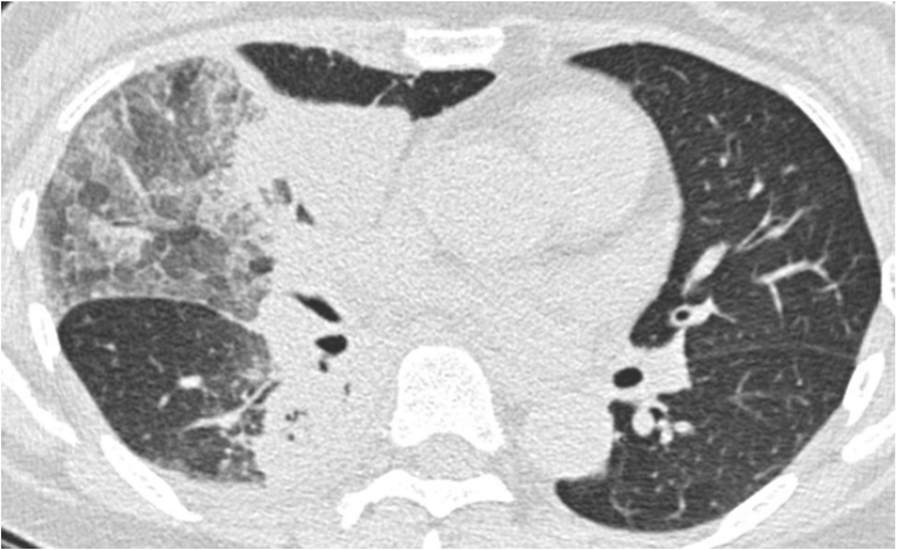
Computed tomography image of the chest showing infiltration in the right middle and lower lobes

**Fig. 2:**
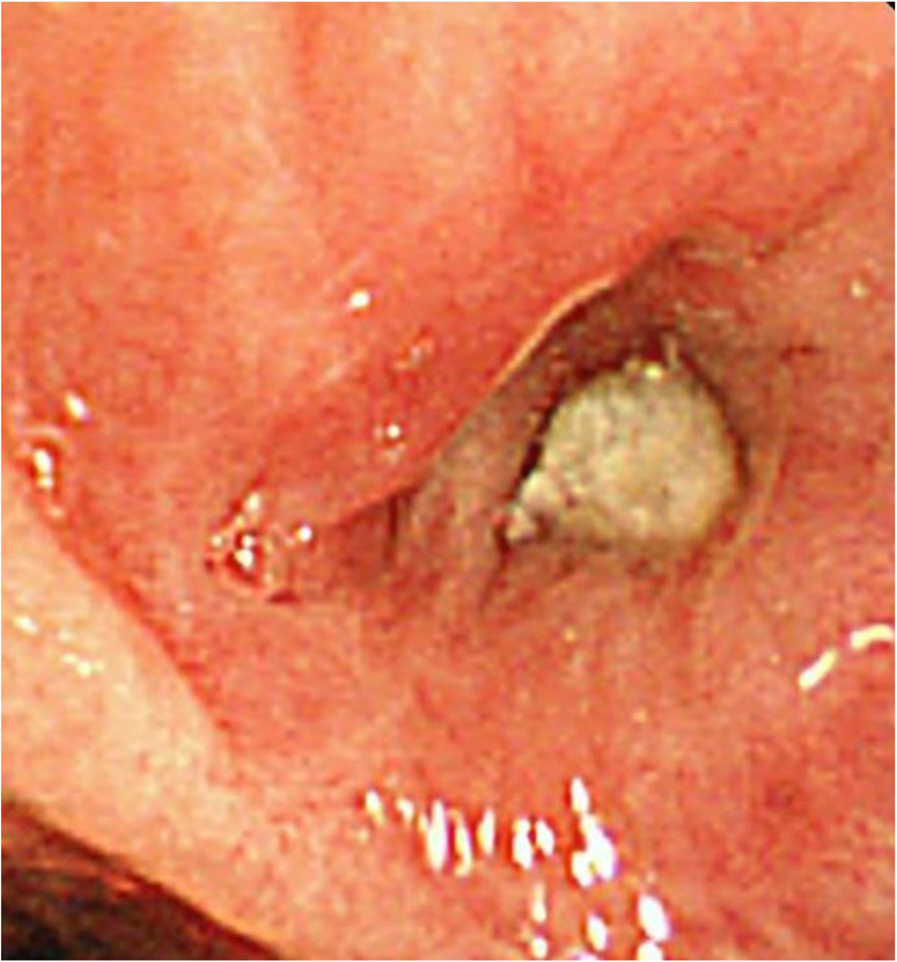
Bronchoscopic findings showing mucus plugging within the right middle lobe bronchus

A 64-year-old woman was diagnosed with bronchial asthma at 40 years of age. Initially, her symptoms were mild and controlled with a moderate dose of inhaled corticosteroid (ICS) and a short-acting beta-agonist. She reported a medical history of eosinophilic rhinitis. At 60 years of age, she experienced frequent wheezing exertion; spirometry revealed forced expiratory volume in 1 s (FEV_1_)/forced vital capacity (FVC) of 62.6%, FEV_1_ of 0.92 L, and peak expiratory flow of 3.37 L/s. She was diagnosed with severe bronchial asthma and treated with an ICS/long-acting beta-agonist (LABA), a long-acting muscarinic antagonist (LAMA), a leukotriene receptor antagonist (LTRA), and theophylline. The patient exhibited purulent sputum, despite these medications, for 3 months and was referred to our hospital in 2013 because of persistent fever that had lasted 2 weeks. Laboratory examination showed a white blood cell count of 12,700/μL (27% eosinophils), C-reactive protein level of 11.79 mg/dL, serum IgE level of 3400 IU/mL, and positive *Aspergillus*-specific IgE (6.24 UA/mL). Serum proteinase-3 anti-neutrophil cytoplasmic antibody (ANCA) and myeloperoxidase-ANCA were negative (< 1.0 U/mL). Precipitating antibody to *Aspergillus* was positive by the technique of Ouchterlony. A chest radiograph showed opacity in the right lower field; a computed tomography (CT) scan showed infiltration in the right middle and lower lobes, as well as mucoid impaction in both lower lobes (Fig. 1). Bronchoscopy revealed a mucoid impaction in the right middle lobe bronchus (Fig. 2); the differential specimen count obtained by bronchial washing from the right middle lobe was composed of 38% eosinophils. Notably, the bronchial washing culture yielded no bacteria or fungus. The patient was diagnosed with ABPA based on the history of bronchial asthma, elevated total IgE, the presence of serum precipitating antibody and elevated specific IgE antibody to *A. fumigatus*, and pulmonary infiltration. Systemic corticosteroid therapy (prednisone 30 mg/day) was initiated, and the patient’s symptoms dissipated. The corticosteroids were gradually tapered, then discontinued after 3 months. The patient developed “moon face” as a consequence of corticosteroid treatment; peripheral eosinophilia and pulmonary infiltration appeared again, 5 months after the cessation of corticosteroid treatment. Systemic corticosteroids (prednisone 10 mg/day) were re-initiated, and itraconazole (200 mg/day) was added as an anti-fungal agent. Systemic corticosteroid treatment was again tapered and discontinued after 4 weeks. The patient was free of corticosteroids, aside from treatment with a short course of systemic corticosteroids for asthma exacerbation, and clinically stable with itraconazole and asthma treatments for 3 years.

**Fig. 3:**
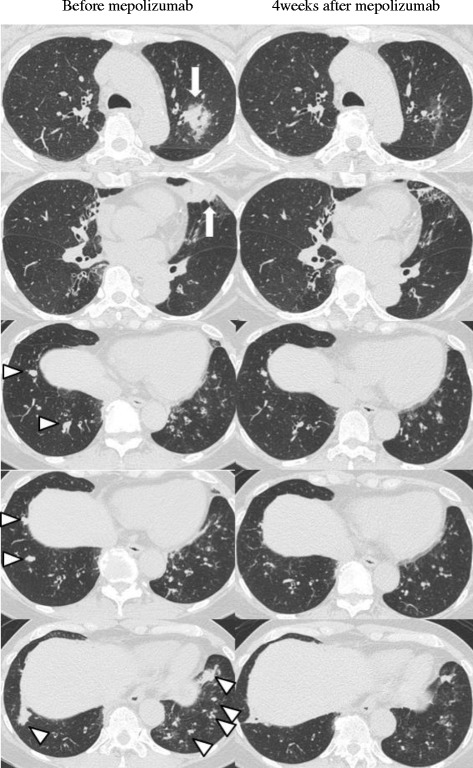
High resolution computed tomography image of the chest showing pulmonary opacities (arrows) in the left upper lobe and mucus plugging (arrow heads) within both lower lobes, prior to mepolizumab treatment. These opacities and mucus plugging were attenuated at 4 weeks after mepolizumab treatment

In 2017, the patient experienced significant deterioration. She complained of wheezing, productive cough, and dyspnea on effort. Laboratory examination revealed marked peripheral eosinophilia (3017/μL) and a chest CT scan demonstrated pulmonary infiltration in the left upper lobe, as well as mucoid impaction in both lower lobes (Fig. 3). The patient was treated with high-dose ICS/LABA, LAMA, LTRA, and theophylline; spirometry showed severe airway obstruction (FEV_1_/FVC: 66.9%; FEV_1_: 1.01 L). An uncontrolled asthma state was indicated by an Asthma Control Test (ACT) score of 18. Mepolizumab (100 mg every 4 weeks) was initiated for the treatment of severe bronchial asthma with ABPA exacerbation. Bronchial asthma symptoms were dramatically improved, and ACT score was increased to 24 at 4 weeks after mepolizumab treatment. Peripheral eosinophil count decreased from 3017/μL to 230/μL and 174/μL, 1 and 4 weeks after mepolizumab, respectively. Spirometry showed improvement of lung function (FEV_1_/FVC: 69.8%; FEV_1_: 1.25 L after 1 week; FEV_1_/FVC: 69.2%, FEV_1_: 1.28 L after 4 weeks). The serum level of IgE did not change after mepolizumab. A chest CT scan demonstrated the disappearance of pulmonary infiltration and mucoid impaction (Fig. 3).

## Discussion and conclusions

To our knowledge, this is the first case of ABPA to be treated with mepolizumab. Dramatic improvements were observed in symptoms, lung function, peripheral eosinophil counts, and chest images.

Our patient was diagnosed with ABPA because the observed clinical, radiologic, and laboratory findings met essential criteria for ABPA that were proposed in 2013, which include: 1) predisposing conditions: asthma or cystic fibrosis; 2) obligatory criteria: total baseline serum IgE > 1000 IU/mL, as well as positive immediate hypersensitivity skin test or elevated specific IgE to *A. fumigatus*; 3) supportive criteria: eosinophilia > 500 cells/μL, serum precipitating or IgG antibodies to *A. fumigatus*, and consistent radiologic opacities [1]. The radiologic features include transient (consolidation, nodules, and tram-track or gloved-finger appearance) or permanent (bronchiectasis or fibrosis) pulmonary opacities. Although persistent fever, which was seen in our case, is not a common symptom of ABPA, fever is included as one of the symptoms of ABPA [1, 3]. Differential diagnoses in our case included eosinophilic pneumonia and eosinophilic granulomatosis with polyangiitis. The predominant patterns of CT findings in acute eosinophilic pneumonia are consolidation and/or ground-glass opacity, frequently accompanied by interlobular septal thickening [4]. Positive IgE against *A. fumigatus* and mucoid impaction (as documented by imaging and bronchoscopy) suggested ABPA, rather than acute eosinophilic pneumonia, in our case. The absence of extrathoracic manifestation and negative ANCA excluded the possibility of eosinophilic granulomatosis with polyangiitis.

ABPA is a severe type of allergic asthma that occurs in approximately 10% of patients with severe asthma. Although a combination of systemic corticosteroids and anti-fungal agents is a standard therapy, there is a risk of serious side effects with long-term use of systemic corticosteroids, such as “moon face,” immunosuppression, diabetes, gastric ulcer, and osteoporosis. Omalizumab, a monoclonal antibody against IgE, has been used in ABPA treatment [5]. Reviews of ABPA cases, including 17 bronchial asthma cases that were treated with omalizumab, have shown beneficial effects such as reduced symptoms, decreased exacerbation rates, and corticosteroid-sparing effects [6]. The dose of omalizumab is determined by a patient’s baseline total IgE level and body weight. The upper limit of the total IgE level is 1500 IU/mL; omalizumab can be administered up to a maximum dose of 600 mg every 2 weeks [7]. Notably, many patients with ABPA exceed the current dosing parameters of omalizumab because of their high total IgE levels, which is a limitation of omalizumab therapy [8].

Mepolizumab is a monoclonal antibody against interleukin-5, a cytokine that releases eosinophils from bone marrow and activates their functions [9]. Mepolizumab has been shown to reduce the frequency of asthma exacerbations in patients with severe eosinophilic asthma [10]. Moreover, mepolizumab exhibited a corticosteroid-sparing effect in eosinophilic asthma patients who require daily systemic corticosteroids [11]. A post hoc analysis showed that mepolizumab was effective for patients, regardless of prior history of omalizumab use; moreover, most patients in the prior omalizumab use subgroup reported that omalizumab was ineffective [12]. Total IgE level at the start of therapy does not affect the efficacy or adverse effects of mepolizumab, and mepolizumab is recommended as one of the therapeutic options in cases of severe eosinophilic asthma with total IgE > 1500 IU/mL [13].

The mechanisms underlying ABPA exacerbation are complex. The increased secretion of interleukin-4 and interleukin-5 from peripheral cells from patients with ABPA suggests that TH2 inflammation contributes to the pathogenesis of ABPA [14]. Notably, elevated levels of IgE and specific antibody against *A. fumigatus* suggested that clinical benefits may result from treatment with omalizumab; additionally, a marked eosinophilia in peripheral blood and bronchial washing suggested that beneficial effects may result from treatment with mepolizumab. The synergistic effects of omalizumab and mepolizumab have been reported in a patient with severe and steroid-dependent ABPA [15]. Our case showed that a single dose of mepolizumab alone induced a 6-point increase in ACT score, 270-mL increase in FEV_1,_ and a 94% reduction in peripheral eosinophil counts; moreover, it attenuated pulmonary infiltration and mucoid impaction. An ACT score of ≤19 indicates uncontrolled asthma and a 3-point change in ACT score is clinically significant [16]. The improvement of lung function in our case appears to be dramatic, compared with the study of patients with severe eosinophilic asthma, in which the mean increase in FEV_1_ was 100 mL after mepolizumab therapy [10].

It has been reported that an attempt to discontinue omalizumab resulted in exacerbation, which was resolved with reinstitution of omalizumab [6]. In our case, we have maintained mepolizumab as treatment for severe asthma with ABPA. Further observation is necessary to determine the optimal period of mepolizumab treatment.

In this report, we described a case of severe bronchial asthma with ABPA that was successfully treated with mepolizumab. Mepolizumab could serve as an alternative treatment with the potential for systemic corticosteroid-sparing effects. Double-blind, placebo-controlled trials are necessary to establish the efficacy and safety of this novel therapeutic intervention for ABPA.

## Abbreviations

ABPA: Allergic bronchopulmonary aspergillosis
ACT: Asthma control test
ANCA: Anti-neutrophil cytoplasmic antibody
CT: Computed tomography
FEV_1_: Forced expiratory volume in 1 s
FVC: Forced vital capacity
ICS: Inhaled corticosteroid
LABA: Long-acting beta-agonist
LAMA: Long-acting muscarinic antagonist
LTRA: Leukotriene receptor antagonist
